# Comparative analysis of different stroke subtype burden and future trends over 15 years in China and globally from 1990 to 2021

**DOI:** 10.3389/fneur.2025.1631775

**Published:** 2025-07-23

**Authors:** Zeyan Zhang, Sijia Wu, Tianyu Jin, Zhixuan Duan, Xiaoxia Du

**Affiliations:** ^1^School of Rehabilitation, Capital Medical University, Beijing, China; ^2^Department of Neurological Rehabilitation, Beijing Bo’ai Hospital, China Rehabilitation Research Center, Beijing, China; ^3^Department of Oral and Maxillofacial-Head and Neck Oncology, Beijing Stomatological Hospital, School of Stomatology, Capital Medical University, Beijing, China; ^4^Department of Rehabilitation Medicine, The Second Affiliated Hospital and Yuying Children's Hospital of Wenzhou Medical University, Wenzhou, Zhejiang, China

**Keywords:** incidence, prevalence, mortality, disability-adjusted life years (DALYs), stroke

## Abstract

**Objective:**

This study aimed to characterize the temporal trends in the burden of various stroke subtypes, including incidence, prevalence, mortality, and disability-adjusted life years (DALYs), across different age and sex groups in China from 1990 to 2021 and compare these trends with global data.

**Methods:**

This study utilizes data from the Global Burden of Disease (GBD) 2021 public dataset to examine temporal trends in different stroke subtypes over the past three decades, both globally and in China. Joinpoint regression and autoregressive integrated moving average (ARIMA) models were applied to analyze historical patterns and forecast future trajectories.

**Results:**

The age-standardized incidence rate (ASIR) and the age-standardized prevalence rate (ASPR) exhibited similar trends, with ischemic stroke (IS) showing an increase, while intracerebral hemorrhage (ICH) and subarachnoid hemorrhage (SAH) displayed a decline. The ASIR of IS in China increased from 100.049 to 135.789 per 100,000. In contrast, the ASIR for ICH decreased from 108.931 to 61.153 per 100,000, and the ASIR for SAH declined from 17.957 to 7.811 per 100,000. In terms of mortality and DLAYs, the age-standardized mortality rate (ASMR) and the age-standardized DALYs (ASDR) for IS, ICH, and SAH all declined. Globally, stroke burden decreased for all subtypes, with men generally more affected than women. The highest burden remains in older populations. Projections suggest a continued rise in IS and ICH prevalence, but a stable trend for SAH.

**Conclusion:**

In China, although the burden of most stroke subtypes has declined, IS and ICH still require sustained prevention and management efforts, particularly in light of their rising prevalence among men and the older adult.

## Highlights

- Previous studies have mostly focused on the analysis of temporal trends in overall stroke burden, failing to differentiate between subtypes in terms of epidemiologic characteristics and disease burden, which may result in a waste of resources. This is the first study to analyze the temporal trends in the burden of stroke subtypes (IS, SAH, and ICH) in China, including incidence, prevalence, mortality, and DALYs.
- The study found that the ASIR and ASPR of IS have been increasing, whereas those of SAH and ICH have been declining.
- However, our study has several limitations. First, we were unable to analyze the disease burden of stroke subtypes across different provinces in China. A more detailed analysis at the provincial level is necessary for a comprehensive understanding. Second, data from certain remote areas may be missing due to the lack of health registry information. Therefore, more extensive health surveys, with greater detail and more representative data from remote regions, are needed.

## Introduction

According to the Global Burden of Disease (GBD) 2021 analysis, stroke is the third leading cause of age-standardized mortality globally, following ischemic heart disease and Coronavirus Disease 2019 (COVID-19) (1), and the fourth leading cause of disability-adjusted life years (DALYs) (2). Ischemic stroke (IS) accounts for approximately 65.3% (95% CI: 62.4–67.7%) of all new stroke cases (3). According to the internationally recognized TOAST classification (4), IS is categorized into five major subtypes: large-artery atherosclerosis, cardioembolism, small-vessel occlusion (also referred to as lacunar infarction), stroke of other determined etiology, and stroke of undetermined etiology. Intracerebral hemorrhage (ICH) comprises 28.8% (95% CI: 28.3–28.8%) of new stroke cases (3), and is primarily caused by rupture of cerebral blood vessels due to hypertension-related degenerative changes or cerebral amyloid angiopathy. Major risk factors include poor blood pressure control and the increased use of anticoagulants, thrombolytics, and antiplatelet agents (5). Subarachnoid hemorrhage (SAH), accounting for only 5.8% (95% CI: 5.7–6.0%) of strokes (3), is characterized by a sudden and severe headache, which is its hallmark symptom. Approximately 80% of SAH cases result from ruptured aneurysms and are associated with a poor clinical prognosis (6). Although stroke incidence, mortality, and DALYs rates for people aged 70 years or older decreased substantially from 1990 to 2021, stroke incidence among those under 70 increased by 4.1% (95% CI: 0.9–7.6), and stroke prevalence in this age group rose by 14.8% (95% CI: 13.1–16.8) (2). This trend may be associated with an aging population and an increasing incidence of stroke in younger individuals (7), contributing to the growing socioeconomic burden on families. The rise in stroke burden may be linked to the limited effectiveness of current primary stroke and cardiovascular disease prevention strategies (8).

GBD 2021 focuses primarily on macro-level assessments at global and regional scales (9). However, as a country with one-fifth of the world’s population (10), the burden of stroke in China has garnered significant attention from the medical community. Previous studies have not specifically analyzed the disease burden of different stroke subtypes in China, but have instead provided a general description of the overall stroke burden (11), which may result in an unequal distribution of healthcare resources. GBD 2021 provides a valuable framework for quantifying the epidemiology and burden of various diseases, including stroke and its subtypes [IHME | GHDx GBD 2021 data resources].^1^ The incidence, prevalence, mortality rates, and DALYs of stroke subtypes exhibit substantial variation (12). Analyzing these subtypes is crucial for obtaining a comprehensive understanding of the most recent trends in stroke, which will aid in healthcare planning, resource allocation, and the prioritization of interventions for different stroke subtypes.

In this study, we assessed the overall burden of IS, ICH, and SAH, including incidence, prevalence, mortality, and DALYs. We also projected the trends in the burden of these stroke subtypes in China and globally over the next 15 years.

## Methods

### Patient and public involvement

It was not appropriate or possible to involve patients or the public in the design, or conduct, or reporting, or dissemination plans of our research.

### Data sources

This study utilized data from the GBD 2021 dataset, which provides a comprehensive analysis of global health losses. GBD 2021 incorporated 100,983 data sources to estimate years of life lived with disability (YLD), years of life lost (YLL), DALYs, and healthy life expectancy (HALE) for 371 diseases and injuries (13). The GBD dataset integrates data from various sources, including cohort studies, randomized controlled trials, national surveys, and other research methods to quantify risks and exposures (14).

Stroke was defined according to the World Health Organization (WHO) clinical criteria as the rapid onset of clinical signs of cerebral dysfunction lasting more than 24 h or leading to death. IS was defined as an episode of neurological dysfunction resulting from focal cerebral, spinal, or retinal infarction. ICH was defined as a stroke accompanied by a focal collection of blood in the brain, not caused by trauma. SAH was defined as a non-traumatic stroke resulting from bleeding into the subarachnoid space of the brain (15).

In this study, data related to stroke subtypes were obtained from the Global Health Data Exchange,^2^ which provides access to the GBD dataset. We used the GBD tool to extract data on the incidence, prevalence, mortality, and DALYs of stroke subtypes in China and globally from 1990 to 2021 as indicators to assess the disease. The specific search parameters were as follows: the selected diseases were “ischemic stroke” “intracerebral hemorrhage” and “subarachnoid hemorrhage,” the selected burden of disease indicators were “incidence,” “prevalence,” “deaths,” and “disability-adjusted life years (DALYs),” the selected regions were “China” and “global,” the years selected were “1990–2021,” the sexes selected were “both,” “male,” and “female,” the age groups selected included “<5 years,” “5–9 years,” “10–14 years,” “15–19 years,” “20–24 years,” “25–29 years,” “30–34 years,” “35–39 years,” “40–44 years,” “45–49 years,” “50–54 years,” “55–59 years,” “60–64 years,” “65–69 years,” “70–74 years,” “75–79 years,” “80–84 years,” “85–89 years,” “90–94 years,” “≥95 years,” and “all ages.” Additionally, “age-standardized” data were also selected. Given that the GBD 2021 data are publicly available, an institutional ethics committee review was not required.

### Statistical analysis

We extracted data on the incidence, prevalence, mortality, and DALYs for IS, ICH, and SAH in China and globally from the GBD database. The data included age-standardized incidence rate (ASIR), age-standardized prevalence rate (ASPR), age-standardized mortality rate (ASMR), and age-standardized DALYs rate (ASDR), as well as crude incidence rate (CIR), crude prevalence rate (CPR), crude mortality rate (CMR), and crude DALY rate (CDR). To investigate temporal trends in different stroke subtypes and assess changes in burden over the past three decades, we performed joinpoint regression analyses using Joinpoint software (National Cancer Institute, Rockville, Maryland, United States) (16). This method identifies points in time where significant changes in trends occur and estimates the annual percentage change between those points. Stratified analyses by sex and age group were also conducted to explore potential differences across populations. Additionally, we applied autoregressive integrated moving average (ARIMA) modeling to forecast future trends in the burden of disease for various stroke subtypes. The Akaike Information Criterion was used to determine the optimal order parameters (p, d, q) for the ARIMA model, providing a more nuanced approach to capturing time series dynamics while accounting for other relevant factors (17). The construction of the ARIMA model involved several steps, including serial smoothing tests, model identification, parameter estimation, model diagnostics, and prediction (18). Based on these predictions, we calculated the ASIR, ASPR, ASMR, and ASDR for IS, ICH, and SAH in China and globally from 2022 to 2036.

Data were analyzed and visualized using R statistical software (version 4.3.3) and Joinpoint software (version 5.1.0). A *p* value of < 0.05 was considered statistically significant.

## Results

### Incidence of different stroke subtypes in China and globally

The number of IS cases in China increased from 761,191 (95% CI: 621,354–937,712) in 1990 to 2,772,053 (95% CI: 2,295,713–3,319,150) in 2021. For ICH, cases grew from 774,012 (95% CI: 644,709–896,197) in 1990 to 1,173,288 (95% CI: 1,003,993–1,330,455) in 2021, whereas SAH cases slightly declined from 150,559 (95% CI: 128,669–176,863) in 1990 to 145,138 (95% CI: 125,425–169,016) in 2021. Globally, however, the number of IS cases increased from 4,151,978 (95% CI: 3,536,772–4,868,150) in 1990 to 7,804,449 (95% CI: 6,719,760–8,943,692) in 2021. The number of ICH cases also rose from 2,358,349 (95% CI: 2,052,149–2,634,480) in 1990 to 3,444,338 (95% CI: 3,053,009–3,812,043) in 2021, and the number of SAH cases increased from 508,789 (95% CI: 441,504-587,616) in 1990 to 697,486 (95% CI: 614,334-795,785) in 2021. Among these stroke subtypes, the ASIR for IS in China rose from 100.049 per 100,000 population (95% CI: 81.521–120.906) in 1990 to 135.789 per 100,000 population (95% CI: 113.255–159.831) in 2021. Meanwhile, the ASIR of ICH decreased from 108.931 per 100,000 population (95% CI: 91.662–124.932) in 1990 to 61.153 per 100,000 population (95% CI: 52.982–69.065) in 2021, and SAH’s ASIR dropped from 17.957 per 100,000 population (95% CI: 15.368–21.121) in 1990 to 7.811 per 100,000 population (95% CI: 6.876–8.950) in 2021. On a global scale, the ASIR for IS decreased from 109.794 per 100,000 population (95% CI: 93.557–127.623) in 1990 to 92.392 per 100,000 population (95% CI: 79.835–105.819) in 2021, the ASIR for ICH fell from 59.489 per 100,000 population (95% CI: 51.410–66.639) in 1990 to 40.834 per 100,000 population (95% CI: 36.201–45.213) in 2021, and the ASIR for SAH declined from 11.691 per 100,000 population (95% CI: 10.222–13.505) in 1990 to 8.326 per 100,000 population (95% CI: 7.339–9.479) in 2021 (Table 1).

**Table 1:** All-age stroke subtypes cases, age-standardized incidence, prevalence, mortality, and DALYs for China and globally in 1990 and 2021.

Disease type	Location	Measure	1990		2021	
			All ages	Age-standardized	All ages	Age-standardized
			*n* (95%CI)	*n* (95%CI)	*n* (95%CI)	*n* (95%CI)
Ischemic stroke	China	Deaths	427,970 (362,335–506,370)	75.22 (64.481–88.232)	1,176,952 (986,876–1,372,707)	64.47 (54.031–74.824)
		DALYs	9,926,125 (8,510,100–11,656,218)	1387.933 (1188.744–1621.395)	70,357,912 (64,329,576–76,007,063)	1180.981 (1009.701–1356.672)
		Prevalence	6,577,205 (5,875,417–7,262,376)	759.201 (675.248–850.306)	20,803,932 (18,615,867–22,995,491)	1018.823 (918.501–1123.352)
		Incidence	761,191 (621,354–937,712)	100.049 (81.521–120.906)	2,772,053 (2,295,713–3,319,150)	135.789 (113.255–159.831)
	Global	Deaths	2,317,112 (2,131,460–2,475,546)	73.149 (66.359–77.943)	3,591,499 (3,213,281–3,888,327)	44.183 (39.294–47.805)
		DALYs	46,176,240 (42,961,948–49,414,586)	1286.306 (1195.192–1376.065)	23,430,411 (19,918,946–26,933,909)	837.36 (763.733–904.977)
		Prevalence	34,668,041 (32,153,637–37,171,588)	849.491 (785.915–913.248)	69,944,885 (64,788,695–75,009,603)	819.47 (760.257–878.705)
		Incidence	4,151,978 (3,536,772–4,868,150)	109.794 (93.557–127.623)	7,804,449 (6,719,760–8,943,692)	92.392 (79.835–105.819)
Intracerebral hemorrhage	China	Deaths	913,023 (784,398–1,064,534)	139.674 (121.088–162.032)	1,322,893 (1,108,046–1,567,711)	68.841 (57.615–81.168)
		DALYs	22,779,117 (19,630,525–26,510,841)	2830.02 (2441.759–3281.074)	79,457,427 (72,748,913–85,480,165)	1351.55 (1129.113–1600.856)
		Prevalence	3,115,040 (2,764,294–3,518,252)	308.405 (274.49–348.285)	4,385,240 (3,892,101–4,906,565)	222.111 (200.091–246.477)
		Incidence	774,012 (644,709–896,197)	108.931 (91.662–124.932)	1,173,288 (1,003,993–1,330,455)	61.153 (52.982–69.065)
	Global	Deaths	2,341,558 (2,184,645–2,505,961)	61.627 (56.952–66.093)	3,308,367 (3,021,075–3,594,725)	39.087 (35.646–42.453)
		DALYs	63,197,955 (59,216,767–67,076,895)	1516.797 (1421.025–1613.261)	27,463,746 (22,839,243–32,676,709)	923.637 (844.826–993.182)
		Prevalence	11,174,315 (10,197,436–12,318,211)	250.233 (227.98–275.117)	16,603,836 (15,159,477–18,183,399)	194.505 (177.99–212.529)
		Incidence	2,358,349 (2,052,149–2,634,480)	59.489 (51.41–66.639)	3,444,338 (3,053,009–3,812,043)	40.834 (36.201–45.213)
Subarachnoid hemorrhage	China	Deaths	189,598 (90,807–249,015)	27.286 (12.808–36.074)	91,802 (66,672–116,215)	4.718 (3.447–5.952)
		DALYs	5,298,115 (2,791,020–6,806,257)	616.838 (315.451–799.171)	10,641,882 (9,398,963–12,121,263)	115.494 (86.862–142.499)
		Prevalence	1,104,538 (961,687–1,242,645)	107.893 (94.596–121.789)	1,323,287 (1,176,081–1,484,052)	68.884 (61.534–76.902)
		Incidence	150,559 (128,669–176,863)	17.957 (15.368–21.121)	145,138 (125,425–169,016)	7.811 (6.876–8.95)
	Global	Deaths	374,887 (270,973–465,035)	9.537 (6.805–11.908)	352,810 (309,015–401,474)	4.185 (3.659–4.761)
		DALYs	12,031,276 (9,409,790–14,507,855)	275.849 (213.223–335.43)	2,296,534 (1,727,442–2,847,370)	125.198 (110.541–142.614)
		Prevalence	4,901,711 (4,420,922–5,394,922)	109.9 (99.053–121.559)	7,852,792 (7,164,804–8,578,767)	92.169 (84.083–100.597)
		Incidence	508,789 (441,504–587,616)	11.691 (10.222–13.505)	697,486 (614,334–795,785)	8.326 (7.339–9.479)

DALYs, disability-adjusted life years.

### Prevalence of different stroke subtypes in China and globally

The number of IS cases in China rose significantly from 6,577,205 (95% CI: 5,875,417–7,262,376) in 1990 to 20,803,932 (95% CI: 18,615,867–22,995,491) in 2021. Similarly, ICH cases increased from 3,115,040 (95% CI: 2,764,294–3,518,252) in 1990 to 4,385,240 (95% CI: 3,892,101–4,906,565), while SAH cases saw a modest rise from 1,104,538 (95% CI: 961,687–1,242,645) in 1990 to 1,323,287 (95% CI: 1,176,081–1,484,052) in 2021. In comparison, globally, the prevalence of IS cases increased from 34,668,041 (95% CI: 32,153,637–37,171,588) in 1990 to 69,944,885 (95% CI: 64,788,695–75,009,603) in 2021. The global prevalence of ICH cases also grew from 11,174,315 (95% CI: 10,197,436–12,318,211) in 1990 to 16,603,836 (95% CI: 15,159,477–18,183,399) in 2021, and the number of prevalent SAH cases increased from 4,901,711 (95% CI: 4,420,922–5,394,922) in 1990 to 7,852,792 (95% CI: 7,164,804–8,578,767) in 2021.

Regarding ASPR, the ASPR for IS in China increased from 759.201 per 100,000 population (95% CI: 675.248–850.306) in 1990 to 1018.823 per 100,000 population (95% CI: 918.501–1,123.352) in 2021. In contrast, the ASPR for ICH declined from 308.405 per 100,000 population (95% CI: 274.490–348.285) in 1990 to 222.111 per 100,000 population (95% CI: 200.091–246.477) in 2021, and the ASPR for SAH dropped from 107.893 per 100,000 population (95% CI: 94.596–121.789) in 1990 to 68.884 per 100,000 population (95% CI: 61.534–76.902) in 2021. On a global level, the ASPR for IS decreased from 849.491 per 100,000 population (95% CI: 785.915–913.248) in 1990 to 819.470 per 100,000 population (95% CI: 760.257–878.705) in 2021. Similarly, the ASPR for ICH declined from 250.233 per 100,000 population (95% CI: 227.980–275.117) in 1990 to 194.505 per 100,000 population (95% CI: 177.990–212.529) in 2021, and the ASPR for SAH fell from 109.900 per 100,000 population (95% CI: 99.053–121.559) in 1990 to 92.169 per 100,000 population (95% CI: 84.083–100.597) in 2021 (refer to Table 1).

### Mortality for different stroke subtypes in China and globally

In China, the number of deaths due to IS increased from 427,970 (95% CI: 362,335–506,370) in 1990 to 1,176,952 (95% CI: 986,876–1,372,707) in 2021. Deaths from ICH rose from 913,023 (95% CI: 784,398–1,064,534) in 1990 to 1,322,893 (95% CI: 1,108,046–1,567,711) in 2021. On the other hand, deaths from SAH decreased from 189,598 (95% CI: 90,807–249,015) in 1990 to 91,802 (95% CI: 66,672–116,215) in 2021. Globally, the number of deaths from IS increased from 2,317,112 (95% CI: 2,131,460–2,475,546) in 1990 to 3,591,499 (95% CI: 3,213,281–3,888,327) in 2021. The number of deaths from ICH rose from 2,341,558 (95% CI: 2,184,645–2,505,961) in 1990 to 3,308,367 (95% CI: 3,021,075–3,594,725) in 2021. Deaths from SAH, however, decreased slightly from 374,887 (95% CI: 270,973–465,035) in 1990 to 352,810 (95% CI: 309,015–401,474) in 2021.

In terms of ASMR, the ASMR for IS in China decreased from 75.22 per 100,000 population (95% CI: 64.48–88.23) in 1990 to 64.47 per 100,000 population (95% CI: 54.03–74.82) in 2021. The ASMR for ICH declined from 139.67 per 100,000 population (95% CI: 121.09–162.03) in 1990 to 68.84 per 100,000 population (95% CI: 57.62–81.17) in 2021. The ASMR for SAH dropped from 27.29 per 100,000 population (95% CI: 12.81–36.07) in 1990 to 4.72 per 100,000 population (95% CI: 3.45–5.95) in 2021. Globally, the ASMR for IS decreased from 73.15 per 100,000 population (95% CI: 66.36–77.94) in 1990 to 44.18 per 100,000 population (95% CI: 39.29–47.81) in 2021. The ASMR for ICH decreased from 61.63 per 100,000 population (95% CI: 56.95–66.09) in 1990 to 39.09 per 100,000 population (95% CI: 35.65–42.45) in 2021. Finally, the ASMR for SAH dropped from 9.54 per 100,000 population (95% CI: 6.81–11.91) in 1990 to 4.19 per 100,000 population (95% CI: 3.66–4.76) in 2021 (see Table 1).

### DALYs for different stroke subtypes in China and globally

In China, the DALYs for IS rose from 9,926,125 (95% CI: 8,510,100–11,656,218) in 1990 to 70,357,912 (95% CI: 64,329,576–76,007,063) in 2021. For ICH, DALYs increased from 22,779,117 (95% CI: 19,630,525–26,510,841) in 1990 to 79,457,427 (95% CI: 72,748,913–85,480,165) in 2021, and for SAH, they increased from 5,298,115 (95% CI: 2,791,020–6,806,257) in 1990 to 10,641,882 (95% CI: 9,398,963–12,121,263) in 2021. Globally, the DALYs for IS decreased from 46,176,240 (95% CI: 42,961,948–49,414,586) in 1990 to 23,430,411 (95% CI: 19,918,946–26,933,909) in 2021, for ICH from 63,197,955 (95% CI: 59,216,767–67,076,895) in 1990 to 27,463,746 (95% CI: 22,839,243–32,676,709) in 2021, and for SAH from 12,031,276 (95% CI: 9,409,790–14,507,855) in 1990 to 2,296,534 (95% CI: 1,727,442–2,847,370) in 2021.

In terms of ASDR, the ASDR for IS in China decreased from 1387.93 per 100,000 population (95% CI: 1188.74–1621.40) in 1990 to 1180.98 per 100,000 population (95% CI: 1009.70–1356.67) in 2021. For ICH, the ASDR decreased from 2830.02 per 100,000 population (95% CI: 2441.76–3281.07) in 1990 to 1351.55 per 100,000 population (95% CI: 1129.11–1600.86) in 2021, while for SAH, the ASDR decreased from 616.84 per 100,000 population (95% CI: 315.45–799.17) in 1990 to 115.49 per 100,000 population (95% CI: 86.86–142.50) in 2021. Globally, the ASDR for IS decreased from 1286.31 per 100,000 population (95% CI: 1195.19–1376.07) in 1990 to 837.36 per 100,000 population (95% CI: 763.73–904.98) in 2021. The ASDR for ICH decreased from 1516.80 per 100,000 population (95% CI: 1421.03–1613.26) in 1990 to 923.64 per 100,000 population (95% CI: 844.83–993.18) in 2021, and the ASDR for SAH decreased from 275.85 per 100,000 population (95% CI: 213.22–335.43) in 1990 to 125.20 per 100,000 population (95% CI: 110.54–142.61) in 2021 (see Table 1).

### Joinpoint regression analysis of stroke subtype burden in China and globally

**Figure 1:**
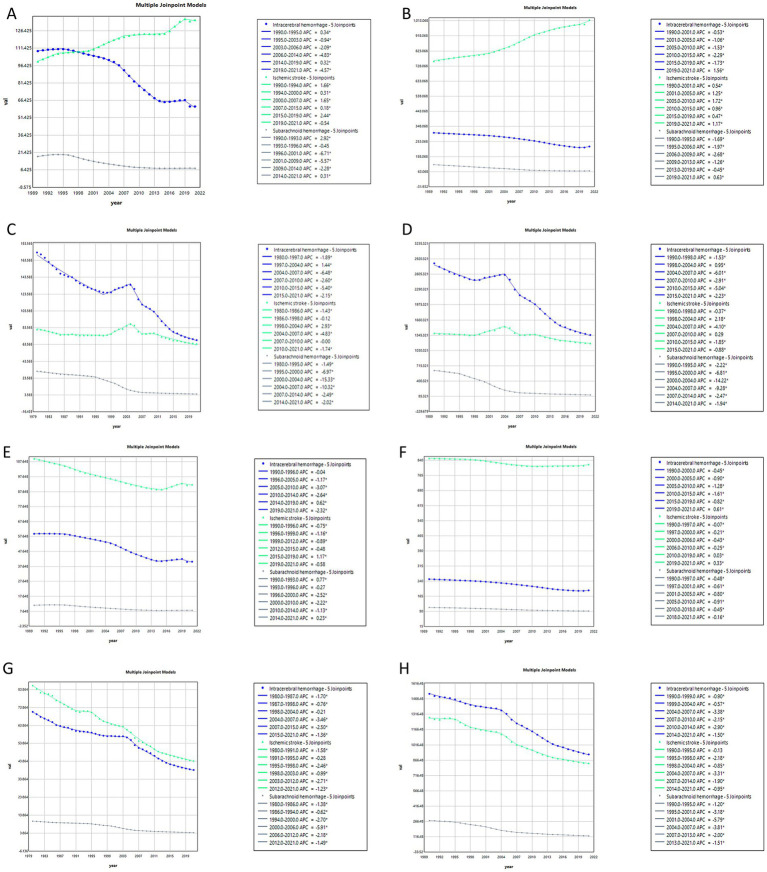
Annual percentage change in age-standardized stroke subtypes burden in China and globally from 1990 to 2021. China: **(A)** age-standardized incidence rate (ASIR); **(B)** age-standardized prevalence rate (ASPR); **(C)** age-standardized mortality rate (ASMR); **(D)** age-standardized disability-adjusted life years (ASDR). Global: **(E)** age-standardized incidence rate (ASIR); **(F)** age-standardized prevalence rate (ASPR); **(G)** age-standardized mortality rate (ASMR); **(H)** age-standardized disability-adjusted life years (ASDR) (^*^indicates *p*-value < 0.05).

The results of the Joinpoint regression analysis for ASIR, ASPR, ASMR and ASDR for different stroke subtypes in China and globally from 1990 to 2021 are presented in Figure 1. In China, the ASIR for ICH showed a significant decline, while the ASIR for IS showed a significant increase. The ASIR for SAH initially increased (annual percent change (APC) = 2.92 from 1990 to 1993), then declined (APC = −0.45 from 1993 to 1996, APC = −6.71 from 1996 to 2001, and APC = −5.57 from 2001 to 2009), before stabilizing with a slight increase from 2009 to 2014 (APC = −2.28) and subsequently rising again from 2014 to 2021 (APC = 0.31). Regarding ASPR, the rate for IS increased significantly, while ASPR for ICH and SAH exhibited a marginal decline. Both the ASMR and ASDR for ICH showed a significant overall decline. The ASMR and ASDR for IS changed relatively smoothly, while those for SAH also exhibited a decreasing trend. Globally, the ASIR for ICH significantly decreased, while that for SAH showed a slight decline. The ASIR for IS initially decreased significantly, then increased slightly (APC = 1.17 from 2015 to 2019), and finally decreased again (APC = −0.58 from 2015 to 2019). The ASPR for ICH, IS, and SAH showed slight decreases with relatively smooth trends. Finally, both the ASMR and ASDR for ICH, IS, and SAH exhibited significant reductions.

### Gender differences in stroke subtype burden in China and globally from 1990 to 2021

**Figure 2:**
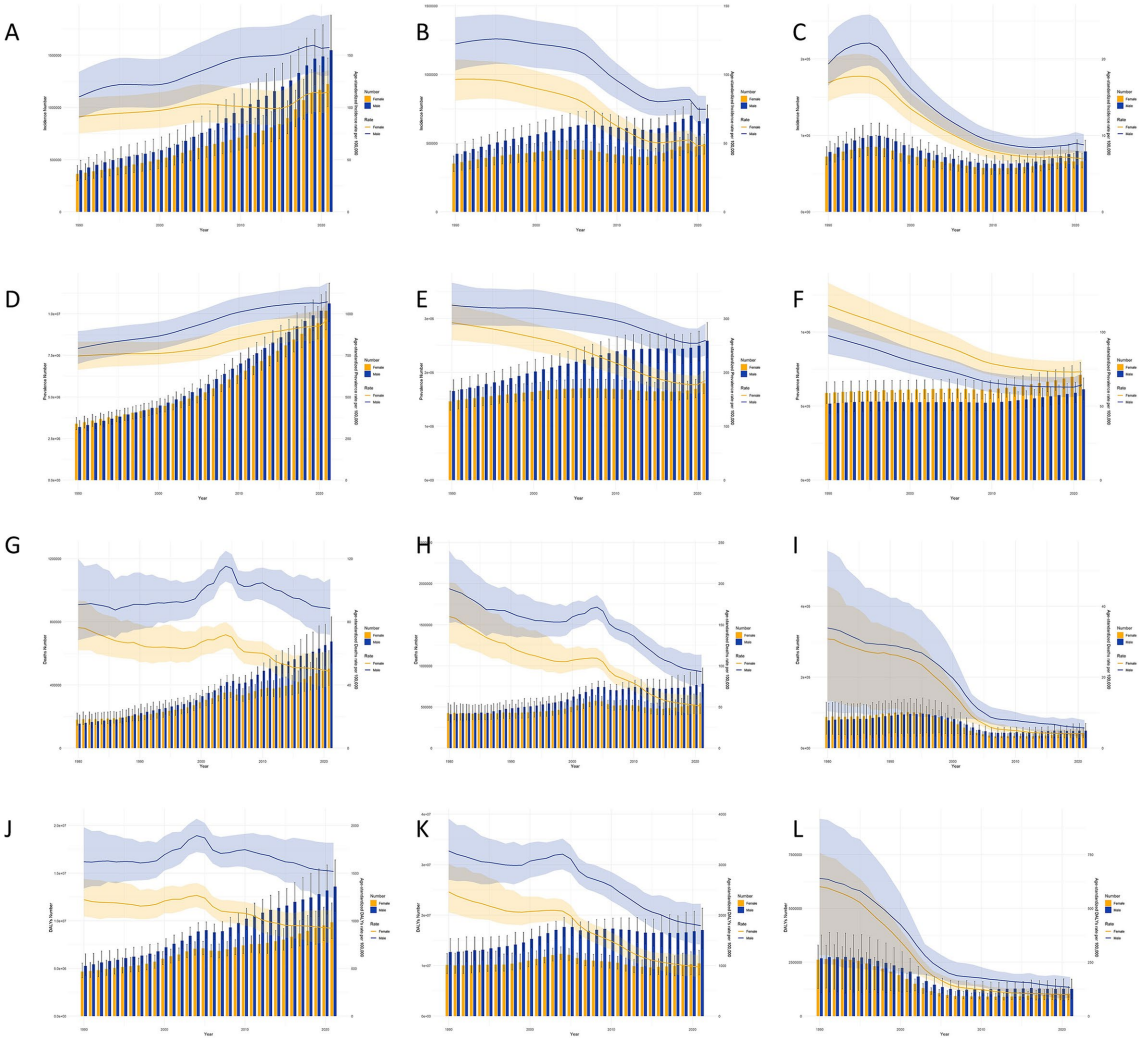
Stroke subtypes cases and age-standardized incidence, prevalence, mortality, and DALYs by Gender in China, 1990–2021. Cases and age-standardized incidence rate (ASIR): **(A)** Ischemic stroke; **(B)** Intracerebral hemorrhage; **(C)** Subarachnoid hemorrhage; cases and age-standardized prevalence rate (ASPR): **(D)** Ischemic stroke; E: Intracerebral hemorrhage; **(F)** Subarachnoid hemorrhage; cases and age-standardized mortality rate (ASMR): **(G)** Ischemic stroke; **(H)** Intracerebral hemorrhage; **(I)** Subarachnoid hemorrhage; DALYs and age-standardized DALYs rate (ASDR): **(J)** Ischemic stroke; **(K)** Intracerebral hemorrhage; **(L)** Subarachnoid hemorrhage. Bar charts represent the number of cases, while line charts show the age-standardized rates.

Figure 2 compares the burden and age-standardized rates of stroke subtypes in men and women in China and globally from 1990 to 2021. From 1990 to 2021, the ASIR for all stroke subtypes was consistently higher in men than in women. Specifically, the ASIR for IS fluctuated upwards, while those for ICH and SAH fluctuated downwards. During this period, the ASPR for IS and ICH remained higher in men than in women, while the ASPR for SAH was higher in women. Notably, the prevalence of IS exhibited a significant upward trend. The ASMR for men was higher than that for women across all stroke subtypes. The ASMR for IS increased, peaked, and then declined after around 2000, while the ASMR for ICH and SAH showed a consistent downward trend. The ASDR followed a pattern similar to the ASMR. In contrast, globally, the ASIR, ASPR, ASMR, and ASDR for all stroke subtypes exhibited a decreasing trend, with higher rates in males than females, except for the ASPR for SAH, which was higher in females.

### Stroke subtype burden across different age groups in China and globally in 1990 and 2021

**Figure 3:**
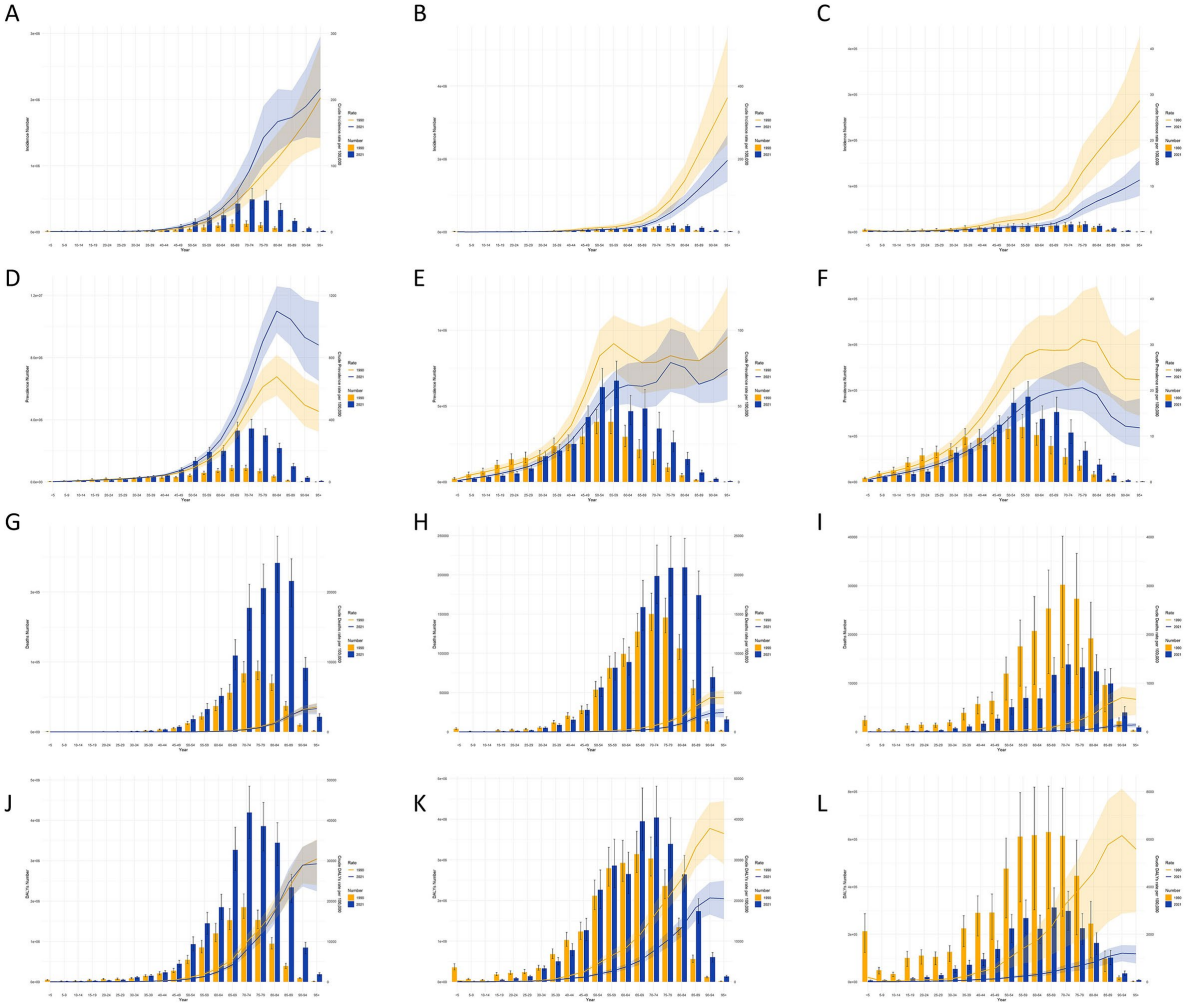
Stroke subtypes cases and age-standardized incidence, prevalence, mortality, and DALYs by age group in china, 1990–2021. Cases and age-standardized incidence rate (ASIR): **(A)** Ischemic stroke; **(B)** Intracerebral hemorrhage; **(C)** Subarachnoid hemorrhage; cases and age-standardized prevalence rate (ASPR): **(D)** Ischemic stroke; **(E)** Intracerebral hemorrhage; **(F)** Subarachnoid hemorrhage; cases and age-standardized mortality rate (ASMR): **(G)** Ischemic stroke; **(H)** Intracerebral hemorrhage; **(I)** Subarachnoid hemorrhage; DALYs and age-standardized DALYs rate (ASDR): **(J)** Ischemic stroke; **(K)** Intracerebral hemorrhage; **(L)** Subarachnoid hemorrhage. Bar charts represent the number of cases, while line charts show the age-standardized rates.

Figure 3 compares the incidence, prevalence, mortality, DALYs, and crude rates (CRs) of stroke subtypes across different age groups in China and globally in 1990 and 2021. In China, IS was most prevalent among individuals aged 65–79 years, with the highest number of cases observed in the 70–74 years group. The highest incidence of ICH and SAH occurred in the 75–79 years group. The CIR showed an increasing trend for IS, which was higher in 2021 compared to 1990, while ICH and SAH had a higher CIR in 1990 than in 2021. This trend was similar globally, although IS CIR was higher globally in 1990 than in 2021. In terms of prevalence, IS peaked in the 70–74 years group in both 1990 and 2021, while ICH and SAH peaked in the 55–59 years group. The CPR for IS and SAH increased and then decreased, while the CPR for ICH fluctuated and increased with age. Globally, CPRs for all stroke subtypes increased, except for SAH, which declined after the 75–79 years group in 1990. In 1990, ICH and SAH caused the most deaths in the 70–74 years group in China, whereas IS was the leading cause of death in the 75–79 years group. By 2021, the highest number of deaths from IS and ICH occurred in the 80–84 years group, while SAH had the highest mortality in the 70–74 years group. The CMR for all stroke subtypes increased in both China and globally. In terms of CDR, IS peaked at 70–74 years group in both 1990 and 2021, SAH peaked at 65–69 years group in both years, and ICH peaked at 65–69 years in 1990 and at 70–74 years in 2021. The CDR for stroke subtypes in China showed an increasing trend up to the 90–94 years group, after which it fluctuated. Globally, CDR also showed an increasing trend.

### Projected trends in the incidence of stroke subtypes over the next 15 years

**Figure 4:**
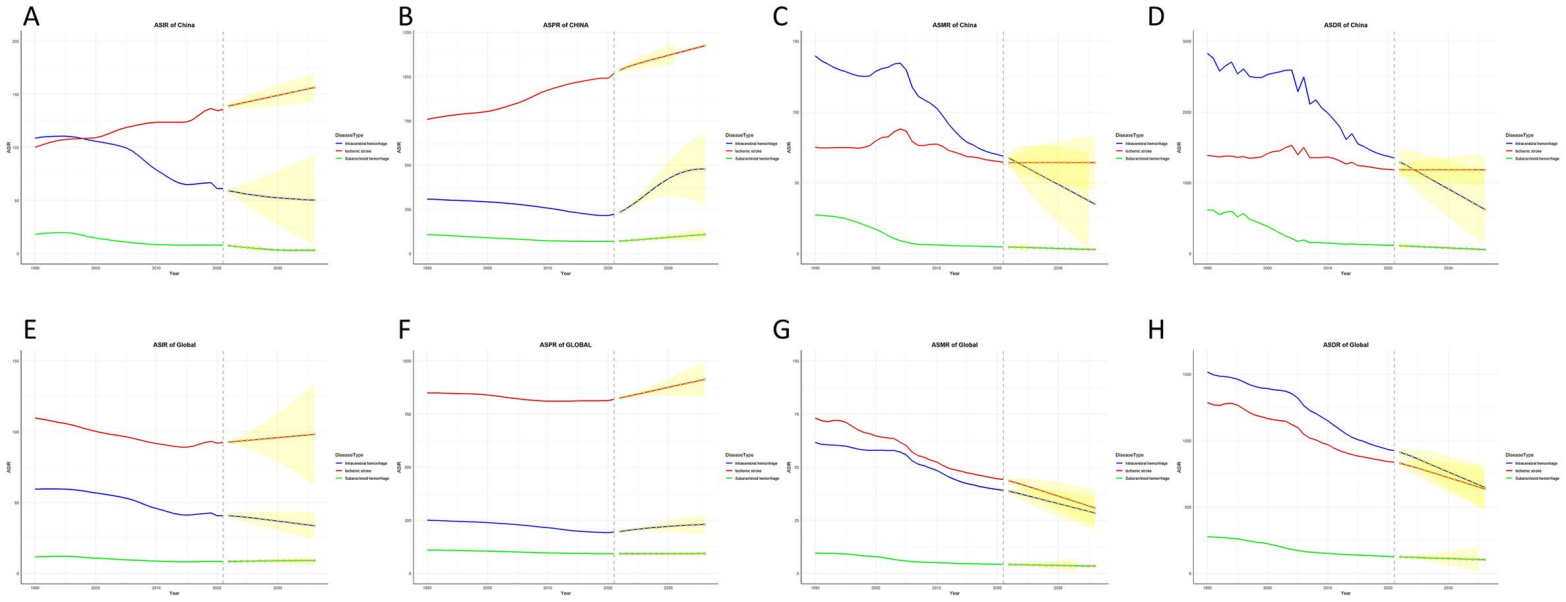
Trends and future projections of stroke subtypes burden in China and globally, 1990–2036. China: **(A)** age-standardized incidence rate (ASIR); **(B)** age-standardized prevalence rate (ASPR); **(C)** age-standardized mortality rate (ASMR); **(D)** age-standardized disability-adjusted life years (ASDR). Global: **(E)** age-standardized incidence rate (ASIR); **(F)** age-standardized prevalence rate (ASPR); **(G)** age-standardized mortality rate (ASMR); **(H)** age-standardized disability-adjusted life years (ASDR) (^*^indicates *p*-value < 0.05). The red line represents Ischemic stroke, the blue line represents Intracerebral hemorrhage, the green line represents Subarachnoid hemorrhage, and the yellow line and shaded area represent projected data.

Figure 4 presents projections of the ASIR, ASPR, ASMR, and ASDR for stroke subtypes in China and globally, based on the ARIMA model. Over the next 15 years, the ASIR and ASPR for IS in China are expected to continue increasing, while the ASMR and ASDR are anticipated to remain stable. Globally, the ASIR and ASPR for IS are projected to increase significantly, while the ASMR and ASDR are expected to continue decreasing. For ICH, the ASIR, ASMR, and ASDR are predicted to decline further, whereas the ASPR is expected to rise significantly. A similar trend is anticipated globally. The projections for SAH indicate that the ASIR, ASPR, ASMR, and ASDR will remain relatively stable, aligning with the global trend.

## Discussion

Using data from the GBD 2021 database, this study provides a comprehensive assessment of the incidence, prevalence, mortality, and DALYs associated with stroke subtypes in China and globally over the past 30 years, along with projections for the next 15 years. We analyzed variations in the burden of stroke subtypes across different age groups and genders in China. The results indicate that, from 1990 to 2021, ICH, including SAH, demonstrated a decreasing trend in ASIR, ASMR, ASPR, and ASDR in China. In contrast, IS showed an increasing trend in ASIR and ASPR, but a decreasing trend in ASMR and ASDR. Globally, the ASIR, ASPR, ASMR, and ASDR for all stroke subtypes exhibited a downward trend. Projections for the next 15 years suggest that the ASIR and ASPR for IS will continue to increase significantly, while the ASPR for ICH is expected to rise, although the ASIR, ASMR, and ASDR are projected to keep declining. For SAH, the burden is expected to remain stable. The burden of stroke subtypes is strongly correlated with age, with higher prevalence and mortality rates observed in individuals aged 65 and older. In terms of gender, men have a higher risk of developing any stroke subtype and are more likely to die from stroke compared to women.

Specifically, with regard to ASIR, global trends show a significant decline in IS, ICH and SAH. In China, however, while the incidence of ICH and SAH has markedly decreased, the incidence of IS continues to rise. This suggests that, aside from IS, the incidence of other stroke subtypes in China has been relatively well controlled-likely as a result of advancements in hypertension prevention and management (19). In terms of prevalence, the number of stroke cases across all subtypes has increased both globally and in China. However, the ASPR for ICH and SAH have declined in both regions. By contrast, both ASIR (100.049 per 100,000 population in 1990 to 135.789 per 100,000 population in 2021) and ASPR (759.201 per 100,000 population in 1990 to 1,018.823 per 100,000 population in 2021) for IS in China have risen substantially, warranting heightened attention. This trend appears to be driven primarily by changes in modifiable risk factors. According to GBD data, elevated systolic blood pressure has remained the leading attributable risk factor for stroke in China since 1990. The burden attributable to ambient particulate matter pollution has also shown a continuous upward trajectory. In men, smoking and alcohol consumption are particularly prominent behavioral risk factors (20). A GBD-based study in Vietnam supports this pattern, indicating that behavioral and environmental risks dominate in males, while metabolic risks are more prominent in females (21). Additionally, the growing burden of IS in China may also be linked to population aging, along with increasing levels of sedentary behavior and unhealthy dietary patterns (22, 23). While China has made notable progress in the prevention and management of various stroke subtypes, further efforts are urgently needed to mitigate the increasing burden of IS. Future strategies should emphasize the control of modifiable risk factors and adopt sex-specific approaches to address differential patterns of exposure and vulnerability.

Although deaths from IS and ICH increased in both China and globally, SAH-related deaths decreased in both regions. In terms of DALYs, IS, ICH, and SAH increased in China, but decreased globally. However, ASMR and ASDR for different stroke subtypes decreased significantly in both China and globally. This improvement may be attributed to factors such as the widespread use of thrombolytic therapy and mechanical thrombectomy (24), better control of cardiovascular risk factors (25), and improvements in public health policies and healthcare interventions (26, 27).

The burden of stroke subtypes in both China and globally showed significant gender differences across various age groups and years. Except for SAH, for which the ASPR was higher in females (73.206 per 100,000 population, 95% CI: 65.604 per 100,000 population–81.486 per 100,000 population) than in males (64.321 per 100,000 population, 95% CI: 57.230 per 100,000 population–71.959 per 100,000 population) in China, the overall disease burden was higher in males. Possible explanations include the influence of estrogen and cerebral aneurysms, the effect of hormones on vascular remodeling, and the higher frequency of vascular wall incompetence in women, which may increase the risk of aneurysms. Additionally, the incidence of cerebral aneurysms is higher in women than in men, particularly after menopause due to hormonal changes (28, 29). The higher ASIR of IS and ICH in males (IS: 157.381 per 100,000 population, 95% CI: 130.602 per 100,000 population–188.870 per 100,000 population, ICH: 74.719 per 100,000 population, 95% CI: 64.165 per 100,000 population–84.315 per 100,000 population) compared to females (IS: 114.934 per 100,000 population, 95% CI: 95.476 per 100,000 population–135.803 per 100,000 population, ICH: 48.205 per 100,000 population, 95% CI: 41.306 per 100,000 population–55.253 per 100,000 population) may be closely associated with a range of behavioral and lifestyle factors. In Asian populations, men demonstrate significantly higher rates of smoking and alcohol consumption compared to women. Notably, chronic heavy alcohol use has been extensively documented as a major risk factor for stroke. A study examining alcohol consumption patterns across various regions in China found that the proportion of male drinkers was substantially higher than that of females, with over 80% of individuals categorized as heavy drinkers being male (30). Furthermore, alcohol intake has been shown to have a positive, approximately linear association with increased risks of stroke, coronary artery disease, heart failure, fatal hypertensive disorders, and fatal aortic aneurysms—further exacerbating the stroke burden among men (31). These findings highlight the critical need for sex-specific stroke prevention strategies that address distinct behavioral risk profiles. Future public health interventions should incorporate targeted, gender-sensitive approaches that reflect these differential patterns of risk exposure.

This study examined the disease burden associated with different stroke subtypes across various age groups from 1990 to 2021. Currently, the peaks of the CIR, CPR, CMR, and CRD for IS, ICH and SAH are concentrated in older age groups, specifically those over 65 years. However, for ICH (667,154, 95% CI: 554,876–795,416) and SAH (185,840, 95% CI: 156,480–219,290), the prevalence peaks have shifted to the 55–59 years group, indicating a trend toward younger populations. This shift may be attributed to higher levels of fasting glucose, body mass index, and red meat intake in younger individuals (32), as well as an increased prevalence of hypertension in these groups (33), all of which elevate the risk of cerebrovascular rupture. Additionally, advancements in medical screening and diagnostic techniques may contribute to earlier detection of strokes in younger patients. These findings highlight the need for stroke prevention and management strategies to move beyond a sole focus on older adults and instead incorporate younger and middle-aged populations. In these groups, early intervention and comprehensive risk factor management are critical. For older adult individuals, current evidence supports the use of integrated care models or stroke unit–based management approaches (34), which typically include tight blood pressure control, antiplatelet therapy, anticoagulation for atrial fibrillation, lipid-lowering treatments, and structured physical rehabilitation programs. These measures have been shown to improve functional outcomes and reduce the risk of recurrent stroke (35). Although specific guidelines for stroke prevention in younger adults are lacking, existing studies underscore the importance of targeting lifestyle-related risk factors, particularly poor dietary habits and physical inactivity. Therefore, we recommend strengthening both primary and secondary prevention strategies in this population, including regular cardiovascular risk screening, lifestyle modification programs, and early pharmacological interventions, to help curb the rising incidence of stroke among younger adults (36).

Over the next 15 years, both China and the global community must focus on IS and ICH. The ASIR and ASPR for IS are expected to continue rising in both China and globally, indicating the need for sustained efforts in cardiovascular disease prevention. However, the ASMR and ASDR remain more stable, suggesting that the ongoing progress in current treatment methods (such as thrombolytic therapy, mechanical thrombolysis, and anticoagulation) has yielded some positive results (37). The ASIR, ASMR, and ASDR for ICH have remained stable in both China and globally, but the ASPR is projected to increase, underscoring the need for enhanced management of hypertension, especially among the older adult and high-risk populations. This highlights the importance of large-scale hypertension screening to ensure broader access to appropriate treatment. Current prevention and treatment efforts for SAH have shown encouraging results.

Our analysis has several limitations. First, the validity and generalizability of the GBD dataset may be limited for rural and underrepresented populations in China, where primary data are often scarce. As a result, the estimates rely heavily on statistical modeling and imputation techniques, which may introduce uncertainty, particularly in areas with limited surveillance capacity. Second, The ARIMA model employed in GBD projections is based on the assumption that historical trends will persist into the future, without incorporating external influences such as policy changes, new health interventions, or socio-environmental shifts. Additionally, it often lacks explicit quantification of forecast uncertainty, which may limit the reliability of long-term predictions. Nonetheless, our study has several notable strengths. To our knowledge, this is the first comprehensive assessment of the burden of stroke subtypes in China, with age- and sex-specific analyses that offer targeted recommendations for prevention and control. Despite the inherent limitations, the GBD data used in this study are of high quality and internationally recognized as a reliable resource for public health research and policy development.

## Conclusion

Based on our findings, future stroke prevention efforts in China should adopt stratified, evidence-based strategies that align with the country’s evolving epidemiological profile. In particular, middle-aged men aged 55–59 should be prioritized through the implementation of nationwide hypertension screening programs and the promotion of behavioral interventions, including smoking cessation and alcohol reduction. These efforts should also address lifestyle-related risk factors, such as unhealthy diets, physical inactivity, and obesity. For women aged 60 and above, increasing awareness and facilitating early detection of SAH through postmenopausal cerebrovascular risk assessments are essential. Additionally, stroke education campaigns should be tailored according to sex and age group to enhance public engagement and adherence to prevention strategies. Effectively addressing these modifiable risk factors is critical for reversing the rising burden of IS and for sustaining long-term management of both ICH and SAH.

## Data Availability

The datasets presented in this study can be found in online repositories. The names of the repository/repositories and accession number(s) can be found at: https://vizhub.healthdata.org/gbd-results/.

## Footnotes

^1^ https://ghdx.healthdata.org/gbd-2021

^2^ https://vizhub.healthdata.org/gbd-results/
