# Supplementary material for: Comparative analysis of different stroke subtype burden and future trends over 15 years in China and globally from 1990 to 2021
Source: Front Neurol. 2025 Jul 23;16:1631775. doi: 10.3389/fneur.2025.1631775 (PMC12327395; doi:10.3389/fneur.2025.1631775)
Supplement: Supplementary file 1 [file Supplementary_file_1.docx]

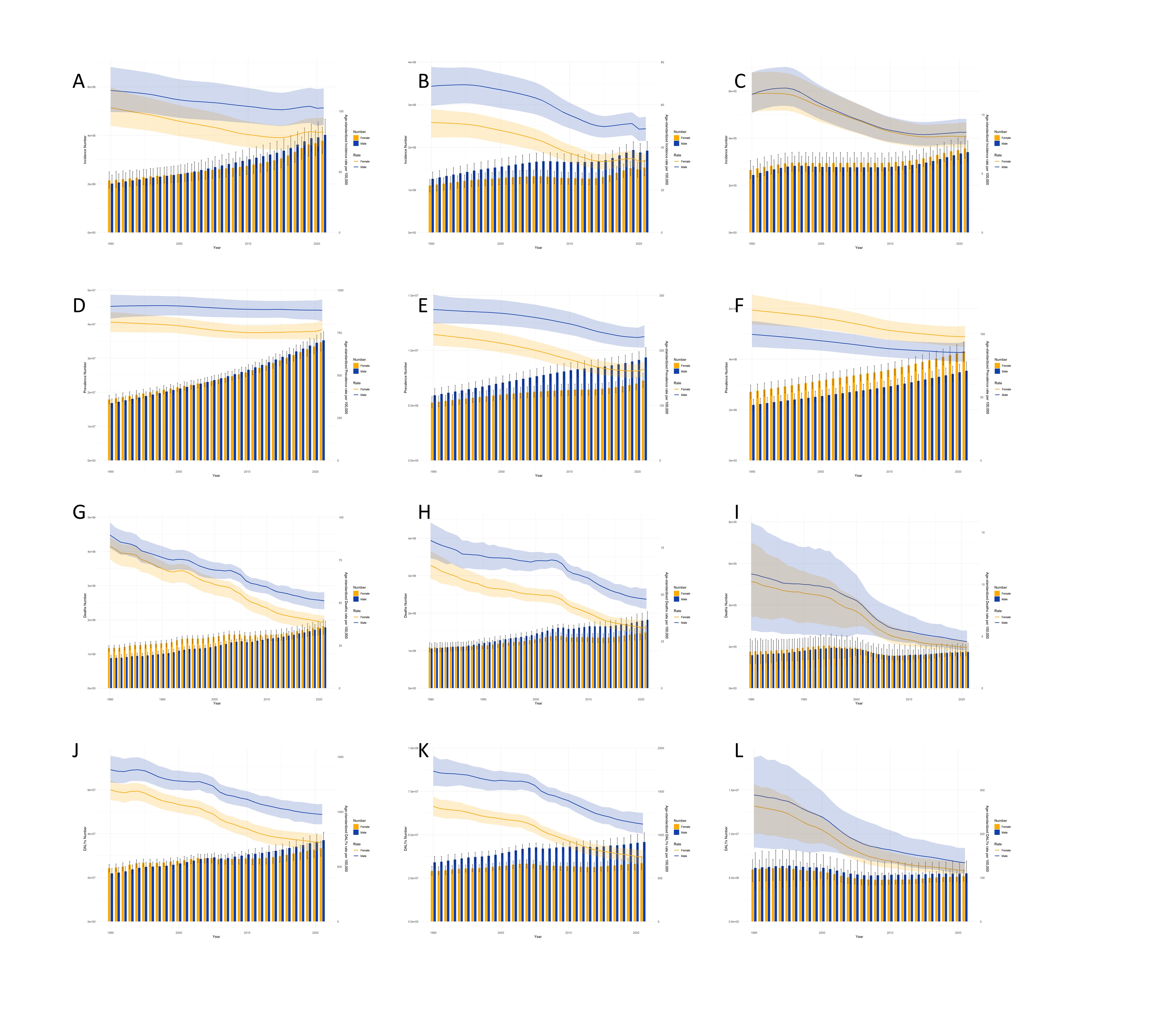
Figure S1. Stroke subtypes Cases and Age-Standardized Incidence, Prevalence, Mortality, and DALYs by Gender in globally, 1990–2021

Cases and Age-Standardized Incidence Rate (ASIR): A: Ischemic stroke; B: Intracerebral hemorrhage; C: Subarachnoid hemorrhage;

Cases and Age-Standardized Prevalence Rate (ASPR): D: Ischemic stroke; E: Intracerebral hemorrhage; F: Subarachnoid hemorrhage;

Cases and Age-Standardized Mortality Rate (ASMR): G: Ischemic stroke; H: Intracerebral hemorrhage; I: Subarachnoid hemorrhage;

DALYs and Age-Standardized DALYs Rate (ASDR): J: Ischemic stroke; K: Intracerebral hemorrhage; L: Subarachnoid hemorrhage. Bar charts represent the number of cases, while line charts show the age-standardized rates.


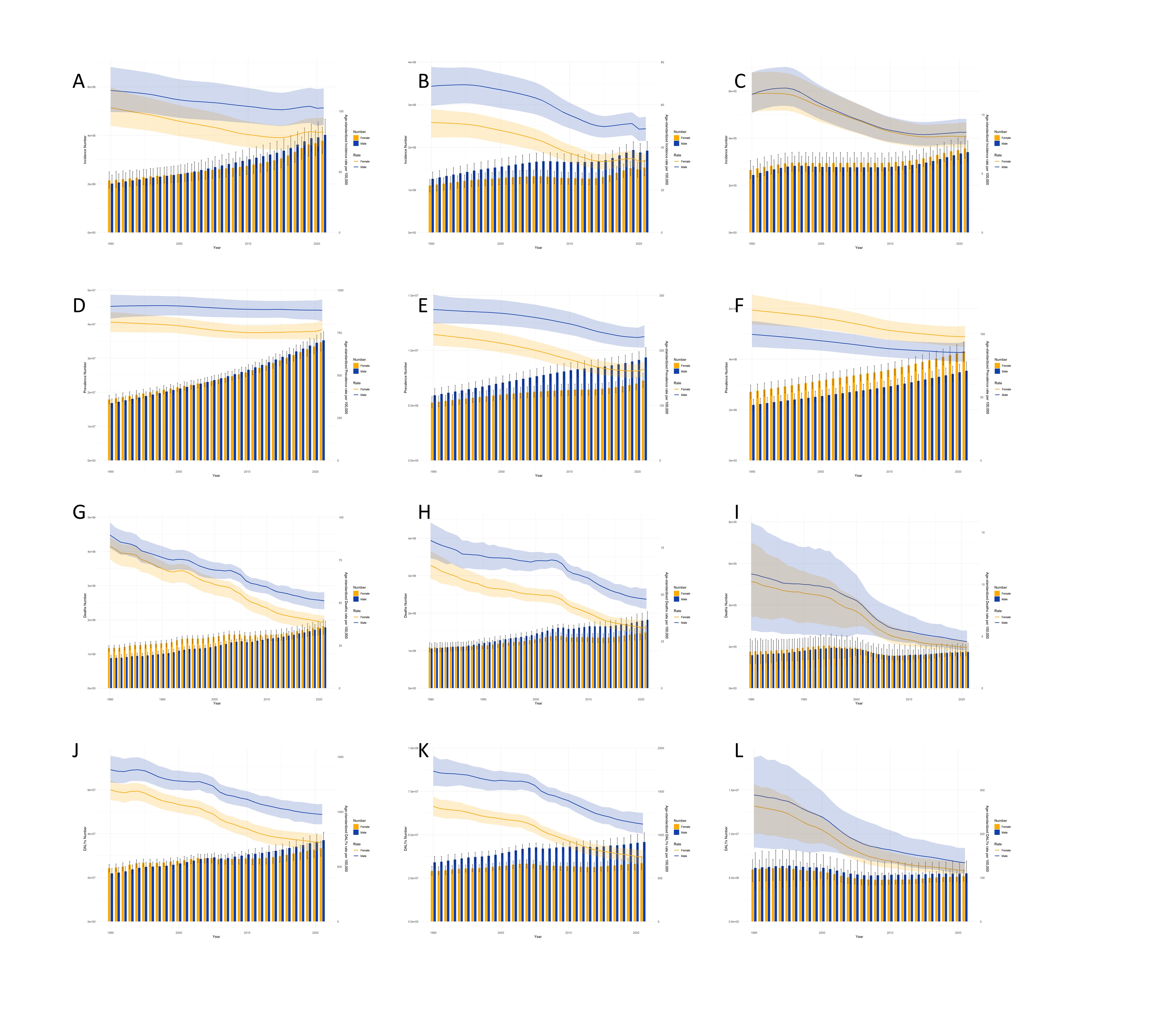
Figure S2. Stroke subtypes Cases and Age-Standardized Incidence, Prevalence, Mortality, and DALYs by Age Group in globally, 1990–2021

Cases and Age-Standardized Incidence Rate (ASIR): A: Ischemic stroke; B: Intracerebral hemorrhage; C: Subarachnoid hemorrhage;

Cases and Age-Standardized Prevalence Rate (ASPR): D: Ischemic stroke; E: Intracerebral hemorrhage; F: Subarachnoid hemorrhage;

Cases and Age-Standardized Mortality Rate (ASMR): G: Ischemic stroke; H: Intracerebral hemorrhage; I: Subarachnoid hemorrhage;

DALYs and Age-Standardized DALYs Rate (ASDR): J: Ischemic stroke; K: Intracerebral hemorrhage; L: Subarachnoid hemorrhage. Bar charts represent the number of cases, while line charts show the age-standardized rates.
